# Intelligent identification on cotton verticillium wilt based on spectral and image feature fusion

**DOI:** 10.1186/s13007-023-01056-4

**Published:** 2023-07-29

**Authors:** Zhihao Lu, Shihao Huang, Xiaojun Zhang, Yuxuan shi, Wanneng Yang, Longfu Zhu, Chenglong Huang

**Affiliations:** 1 College of Engineering, Huazhong Agricultural University, Wuhan, 430070 People’s Republic of China; 2 National Key Laboratory of Crop Genetic Improvement, Hubei Hongshan Laboratory, Huazhong Agricultural University, Wuhan, 430070 People’s Republic of China; 3 Shenzhen Institute of Nutrition and Health, Huazhong Agricultural University, Wuhan, 430070 People’s Republic of China; 4 Shenzhen Branch, Guangdong Laboratory for Lingnan Modern Agriculture, Genome Analysis Laboratory of the Ministry of Agriculture, Agricultural Genomics Institute at Shenzhen, Chinese Academy of Agricultural Sciences, Shenzhen, 518000 People’s Republic of China

**Keywords:** Cotton verticillium wilt, Feature fusion, Hyperspectral, Support vector machine, Back propagation neural networks, Deep learning

## Abstract

**Background:**

Verticillium wilt is the major disease of cotton, which would cause serious yield reduction and economic losses, and the identification of cotton verticillium wilt is of great significance to cotton research. However, the traditional method is still manual, which is subjective, inefficient, and labor-intensive, and therefore, this study has proposed a novel method for cotton verticillium wilt identification based on spectral and image feature fusion. The cotton hyper-spectral images have been collected, while the regions of interest (ROI) have been extracted as samples including 499 healthy leaves and 498 diseased leaves, and the average spectral information and RGB image of each sample were obtained. In spectral feature processing, the preprocessing methods including Savitzky-Golay smoothing (SG), multiplicative scatter correction (MSC), de-trending (DT) and mean normalization (MN) algorithms have been adopted, while the feature band extraction methods have adopted principal component analysis (PCA) and successive projections algorithm (SPA). In RGB image feature processing, the EfficientNet was applied to build classification model and 16 image features have been extracted from the last convolutional layer. And then, the obtained spectral and image features were fused, while the classification model was established by support vector machine (SVM) and back propagation neural network (BPNN). Additionally, the spectral full bands and feature bands were used as comparison for SVM and BPNN classification respectively.

**Result:**

The results showed that the average accuracy of EfficientNet for cotton verticillium wilt identification was 93.00%. By spectral full bands, SG-MSC-BPNN model obtained the better performance with classification accuracy of 93.78%. By feature bands, SG-MN-SPA-BPNN model obtained the better performance with classification accuracy of 93.78%. By spectral and image fused features, SG-MN-SPA-FF-BPNN model obtained the best performance with classification accuracy of 98.99%.

**Conclusions:**

The study demonstrated that it was feasible and effective to use fused spectral and image features based on hyper-spectral imaging to improve identification accuracy of cotton verticillium wilt. The study provided theoretical basis and methods for non-destructive and accurate identification of cotton verticillium wilt.

**Supplementary Information:**

The online version contains supplementary material available at 10.1186/s13007-023-01056-4.

## Background

Cotton is native to tropical and subtropical regions and is a perennial, short-day crop, and it is a strategic material related to the national economy and people’s livelihood. China is with the largest cotton production and consumption in the world, while cotton is also the second largest crop after cereals [1]. Cotton verticillium wilt is a serious disease resulting in yield reduction and economic losses, which is caused by fungal infection through various media such as cottonseed, diseased plant residues, soil, fertilizer, water, and agricultural tools [2]. According to the pathogenicity of the bacteria, there are three types of vert wilt including the deciduous type, withered spot type, and yellow spot type. The disease would occur in the whole growth period of cotton and reach the peak in the flowering and boll-setting period from July to August, and the infected cotton leaves would gradually turn yellow, wither and fall off, which would lead to small cotton bolls and high boll drop rate, and finally result in a decrease in yield and quality [3]. Therefore, the identification of cotton verticillium wilt is of great significance to cotton breeding and genetic research. However, the traditional method is generally manual, and the yellowing, wilting, and death of the cotton leaves were judged and recorded by the eyes observation of the leaf color and shape, which is labor-intensive, subjective, and even destructive [4]. In conclusion, it is necessary to develop an intelligent detection method for cotton verticillium wilt identification.

As an emerging high-precision non-destructive testing technology, hyperspectral imaging technology is widely used in all walks of life, and its application in the agricultural field is particularly important and prominent. Hyperspectral imaging technology is developed by combining spectral and imaging technologies, which can simultaneously obtain spatial and spectral information of objects [5]. Hyperspectral images have the characteristics of “integration of maps”, and also have the advantages of fast, non-destructive and simple, and have become an important research field in the identification and detection of crop diseases, the combination of machine learning and deep learning to establish classification models can accurately identify and detect diseases. Feng et al. [6] developed a hyperspectral imaging system for an accurate prediction of the above-ground biomass of individual rice plants. Linear stepwise regression analysis and fivefold cross-validation were adopted to select valid variables and construct the model. In the tillering to elongation stage, the R^2^ value of fresh weight (FW) was 0.940 and dry weight (DW) was 0.935. In the booting to heading stage, the R^2^ value of FW was 0.891 and DW was 0.783, indicating that hyperspectral imaging is superior to visible light imaging. Pan et al. [7] combined PLS-DA, KNN, and SVM three classification models for pathogenetic process monitoring and early detection of pear black spot disease, and the raw data were processed by three methods: first derivative, MSC, mean centering, while the PCA algorithm was used to extract the feature bands. And it showed that the SVM model had a good effect on early detection of pear black spot disease by hyperspectral technology. Abdulridha et al. [8] obtained hyperspectral images of asymptomatic, early and late infected citrus leaves to classify the diseased leaves in different periods. Pham et al. [9] built a push-broom hyperspectral system to collect hyperspectral image data, and SVM and artificial neural networks models were used to classify surface defects of jujubes online, which can be used for accurate surface defect detection of many other fruits. Gao et al. [10] used hyperspectral imaging to early detect grapevine leafroll disease in a red-berried wine grape cultivar, while squares-SVM was established for classification, and the results indicated that the virus-infected grapevines could be detected during asymptomatic stages with high accuracy. Xuan et al. [11] adopted hyperspectral imaging for early diagnosis and pathogenesis monitoring of wheat powdery mildew, the partial least squares discriminant analysis model obtained the best performances with classification accuracy of 91.4% in validation sets. Lu et al. [12] proposed a spectrum extraction method based on the spots region, while SVM and extreme learning machine (ELM) were established to identify the two similar diseases of tea white star and anthrax disease, and the results showed that the ELM model had the best performance with classification accuracy of 95.77%. The above studies showed that hyperspectral imaging technology combined with traditional machine learning methods has achieved good results in crop disease identification and detection. However, there are few reports about cotton verticillium wilt identification, and this study would verify the feasibility and fill the gap.

In recent years, deep learning has been widely used in agriculture research [13], which has proved powerful image feature extraction ability [14]. Convolutional neural network (CNN) is the most commonly used and prominent network for image feature extraction in deep learning, which acquired general acknowledgement for diverse application areas [15]. Liu et al. [16] used MobileNetV2 model as the primary network to identify and classify six common citrus diseases, which could reduce the model size and keep good classification accuracy. Priyadharshini et al. [17] proposed a deep CNN-based architecture for maize leaf disease classification, which achieved an accuracy of 97.89%. Zhong et al. [18] proposed a novel method to identify apple leaf diseases based on DenseNet-121 deep convolution network, which achieved 93.71% accuracy. While several studies have also been conducted on the cotton phenotype. Jing et al. [19] utilized pre-processed IKONOS imagery to select remote sensing factors for monitoring cotton verticillium wilt, and established a severity estimating model using partial least squares regression analysis. Liang et al. [20] presented a few-shot learning framework for classifying cotton leaf disease spots, comparing various convolutional neural network models to achieve better classification accuracy. Tan et al. [21] proposed two deep learning models (Faster R-CNN and YOLOv5) to detect the number and dehiscence status of cotton anthers, with improved Faster R-CNN demonstrating higher detection accuracy. The above studies demonstrated that deep learning had excellent potential for the image feature extraction, which would provide a new intelligent method for the identification of cotton verticillium wilt, and the feature fusion would explore a novel improvement for the identification of cotton verticillium wilt.

Therefore, this study proposed a novel method for intelligent identification of cotton verticillium wilt based on hyper-spectral imaging by spectral and image feature fusion (FF). Hyperspectral imaging technique was used to obtain spectral and image information, and the spectral data were processed using SG, MSC, DT and MN, then spectral feature bands were extracted using PCA and SPA. In addition, the image features were extracted by EfficientNet. Finally, the extracted spectral and image features were fused for SVM and BPNN classification models. This study would demonstrate a feasible and effective method for cotton verticillium wilt identification with high accuracy.

## Materials and methods

**Fig. 1:**
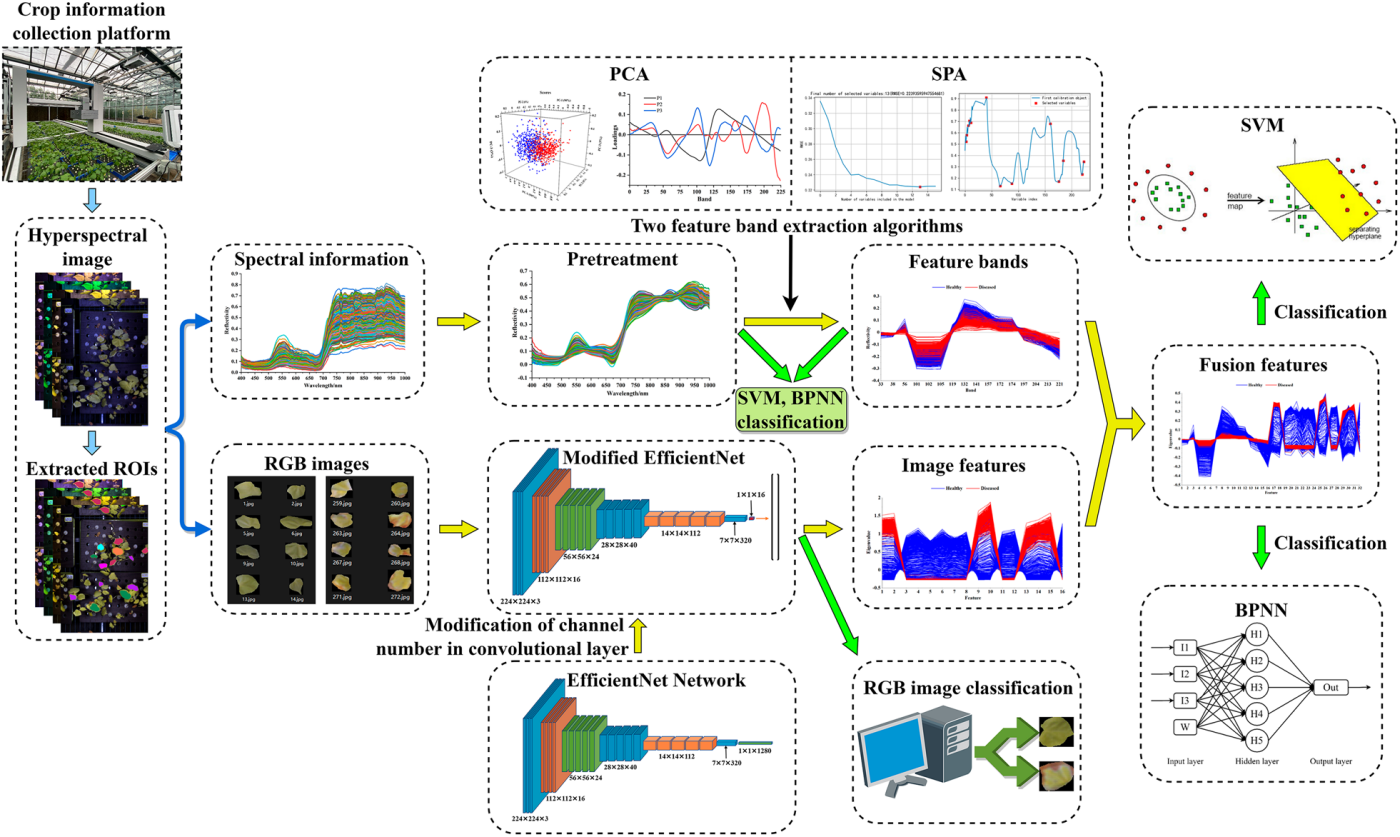
Technical route for cotton verticillium wilt classification by fused features

In this study, the technical route for cotton verticillium wilt classification was shown as Fig. 1. Firstly, the cotton hyperspectral images were obtained by the crop information collection platform, and then the spectral information and RGB image were extracted by ENVI software. Secondly, SVM and BPNN were used to build classification models with the spectral preprocessed full bands, and the feature bands extracted by PCA and SPA, respectively. Thirdly, the modified EfficientNet was utilized to extract the image features from RGB image. Finally, the feature bands and image features were fused for the SVM and BPNN classification of cotton verticillium wilt.

### Cotton materials and information acquisition platform

Sample preparation was conducted at National Key Laboratory of Crop Genetic Improvement at Huazhong Agricultural University, Wuhan, China in June 2021. On June 8th, 2021, 180 pots of different cotton varieties were cultivated in the cotton greenhouse with 9 rows in total, and each row had 20 pots of cotton. The cotton plants were cultivated hydroponically and supplied with a nutrient solution tailored to their specific planting requirements. On June 20, 2021, the cotton was inoculated with verticillium wilt V991. On July 13, 2021, all cotton samples were scanned using the hyperspectral camera of the intelligent information collection platform, and it took about 20 min to scan 9 hyperspectral images in total. 997 cotton leaves were randomly extracted from 180 pots of different cotton varieties, including 499 healthy leaves and 498 diseased leaves.

**Fig. 2:**
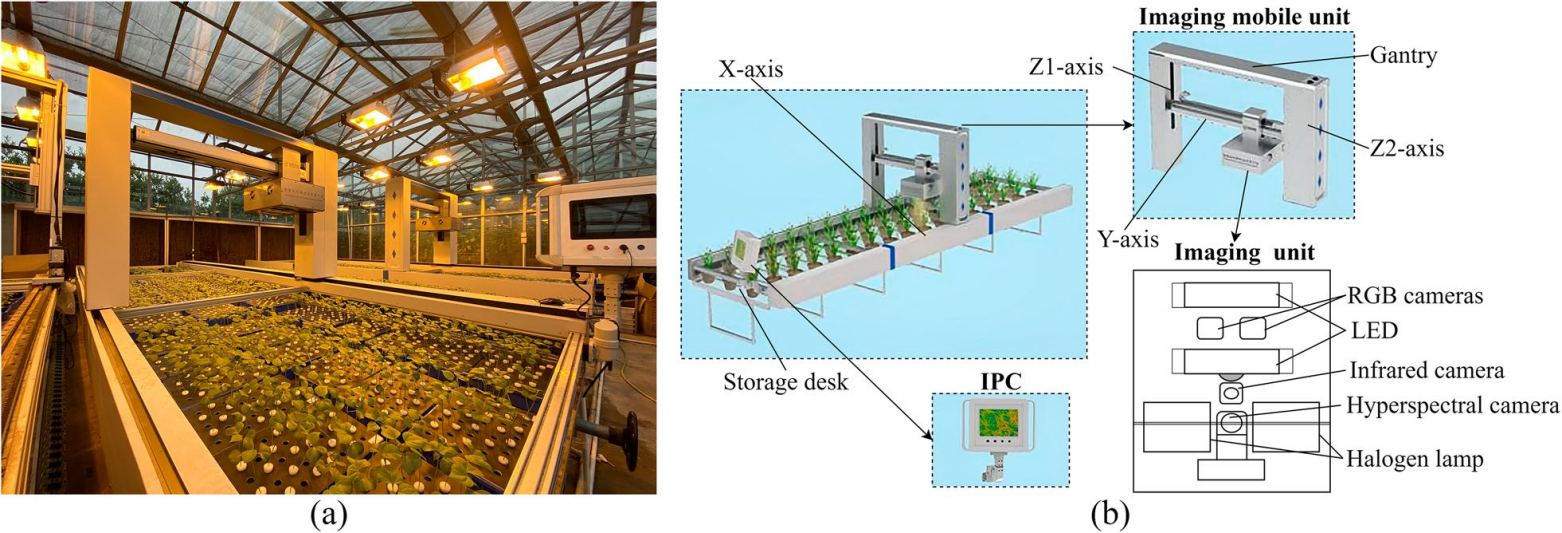
Cotton information acquisition platform on seedbed. **a** Greenhouse application; **b** Structure diagram

The cotton information collection platform was shown as Fig. 2a, and its structural diagram was shown as Fig. 2b, which was mainly composed of the industrial personal computer (IPC), motion units and imaging device. The IPC was used to control the movement of the equipment and the shooting of the camera, as well as to realize the storage of the acquired images. The motion units adopted a gantry structure with three-coordinate motion, in which the maximum travel of each axis is 6100 mm, 950 mm, 500 mm for X, Y, Z axis respectively. The imaging device included visible light camera, infrared camera, and hyperspectral imaging unit. The hyperspectral imaging unit has been utilized in the research with the spectral range 400–1000 nm, the spectral resolution 5.5 nm, the spatial resolution 1024 pixel. The number of spectral bands is 224, and the full band halogen lamp was installed as light source.

### Hyperspectral image acquisition and ROIs extraction

**Fig. 3:**
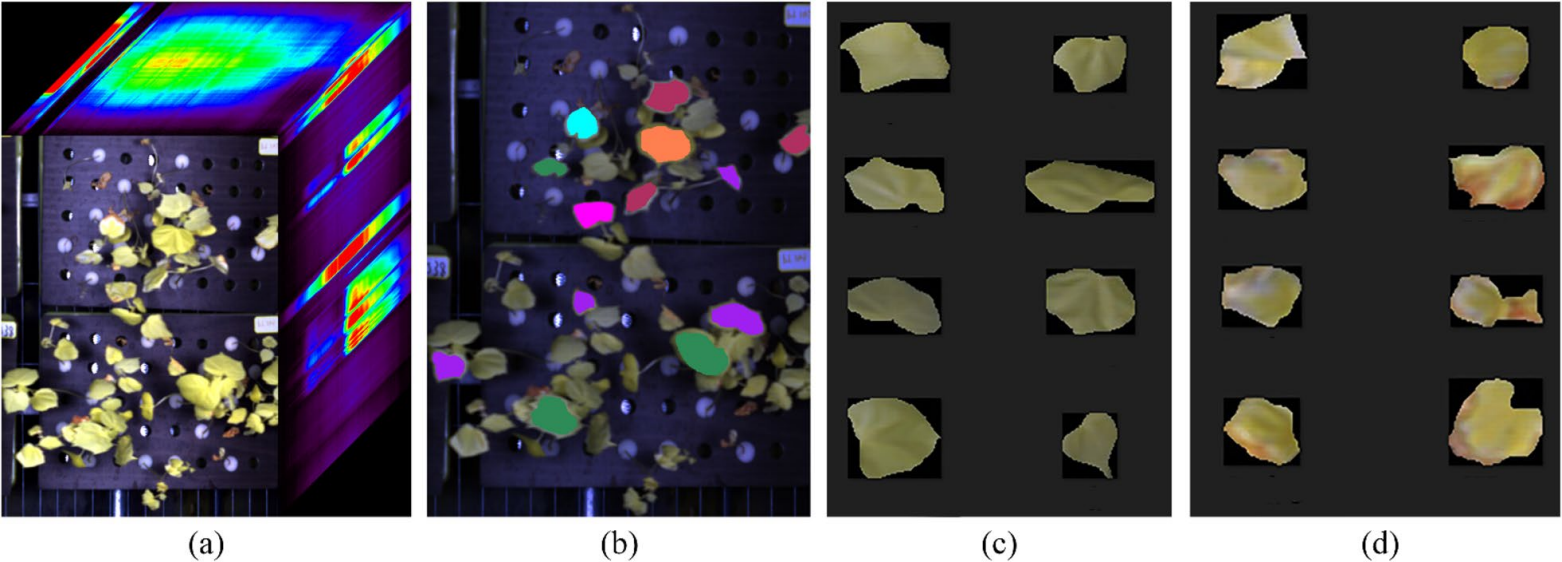
Hyperspectral image acquisition and ROIs extraction, **a** Hyperspectral images, **b** ROIs, **c** RGB images of healthy leaves and **d** diseased leaves

The hyperspectral image acquisition and ROIs extraction was conducted for cotton leaves as shown in Fig. 3. Hyperspectral images of cotton seedlings were automatically collected by controlling the acquisition platform to scan along the X-axis direction. The acquired hyperspectral images, as shown in Fig. 3a, were imported into ENVI software, and the region of interests (ROIs) were manually conducted to extract the cotton leaf as shown in Fig. 3b. The ROIs were extracted based on the shape of the whole leaf, including the leaf borders. A total of 997 ROIs were extracted as research samples, which consisted of 499 healthy leaves and 498 diseased leaves. In order to label the healthy and diseased leaves, three skilled workers made the judgement based on the leave colour and shape. Then the average spectral reflectance and RGB image of each ROI were extracted by ENVI software, and 997 RGB images of leaves were obtained, including healthy leaves (Fig. 3c) and diseased leaves (Fig. 3d).

### Spectral data preprocessing and feature band extraction

In order to eliminate the influence of dark current and uneven illumination, the dark and white calibration were performed for hyperspectral images based on the Eq. 1 [22].

1 $${I}_{c}=\frac{{I}_{raw}-{I}_{dark}}{{I}_{white}-{I}_{dark}}$$

where *I*_*c*_ is the black-and-white corrected cotton hyperspectral image; *I*_*raw*_ is the cotton raw spectral image; *I*_*dark*_ is dark reference image, acquired by covering the lens with an opaque cap; *I*_*white*_ is white reference image, took by scanning the rectangular standard polyethylene white plate.

Due to the influence of environment and hardware, the obtained spectral curve would have some interference such as noise and spectral line drift. Therefore, in order to reduce the influences, the raw hyperspectral data needed to be processed [23]. Savitzky-Golay smoothing (SG) [24] adopts a polynomial to fit local data at each data point and applies the polynomial to smooth the data. Multiplicative scatter correction (MSC) [25] uses a linear regression model to eliminate wavelength shifts caused by sample absorption and scattering under visible light. De-trending (DT) [26] eliminates the interference in spectroscopic data by removing the trend component. Mean normalization (MN) [27] calculates the average value of each wavelength in hyperspectral data and normalize the data to zero-mean and unit variance. In this research, the preprocessing methods including SG, MSC, DT and MN have been adopted by Unscrambler X 10.4 software, and performance of different preprocessing methods would be compared.

The full band spectral data of each sample had 224 spectral bands, which had multi-collinearity redundant information [28]. Full band spectral data was used for model establishment, which would increase the model complexity and decrease the generalization ability of the model, so the dimensionality reduction method was generally applied to extract feature bands for modeling, effectively eliminating irrelevant information and simplifying data. Principal component analysis (PCA) [29] and successive projections algorithm (SPA) [30] were well-established techniques with demonstrated effectiveness in hyperspectral data analysis, which had been widely used in the spectral feature extraction.

PCA is a dimensionality reduction method, which recombines the original variables through orthogonal linear transformation to generate new variables [31]. The original data would be projected into new coordinate system, where the first, second et.al principal components were obtained by the ranking of data variance, to produce mutually orthogonal new variables, and new variables X was decomposed as Eq. 2. With PCA processing, the main features would be extracted, and the collinearity of data variables would be eliminated [32]. SPA is conducted as follows. Firstly, a band was randomly selected from the spectral data randomly, and then the band would be projected to the other remaining bands, while the band with the largest projection would be collected into the band combination. The above steps were repeated to obtain the feature bands, in which the new selected band had the fewest linear relationship with the previous, and finally the optimal feature bands would be refined by model evaluation [33, 34].

2 $$\mathrm{X}={t}_{1}{p}_{1}^{T}+{t}_{2}{p}_{2}^{T}+\cdots +{t}_{n}{p}_{n}^{T}$$

where the vector *t* is the score vector, and the vector *p* is the load vector. Besides $i\ne j$, ${t}_{i}^{T}{t}_{j}$=0, ${p}_{i}^{T}{p}_{j}$=0.

The main configurations of the computer used for data analysis computer was as follows: operating system of Microsoft Windows 10 Enterprise Edition LTSC (64-bit), CPU of 11th Gen Intel(R) Core (TM) i7-11700K @ 3.60GHz (3600 MHz) processor, RAM of 32.00 GB (2133 MHz), and graphics card of NVIDIA GeForce RTX 3060 (12288MB).

### Image feature extraction based on EfficientNet

With rapid development of deep learning algorithms in recent years, a growing number of applications have been reported in agriculture, which had the advantages of adaptive learning and self-feature extraction. Duan et al. [35] proposed a rice panicle segmentation algorithm called PanicleNet based on SegNet, which outperformed the existing crop ear segmentation algorithms. As to image classification, deep learning network would definitely help for the image feature extraction. Therefore, this paper adopted a deep learning network to identify the cotton verticillium wilt with RGB image. Besides the extracted image features and above feature bands were fused for further model improvement.

**Fig. 4:**
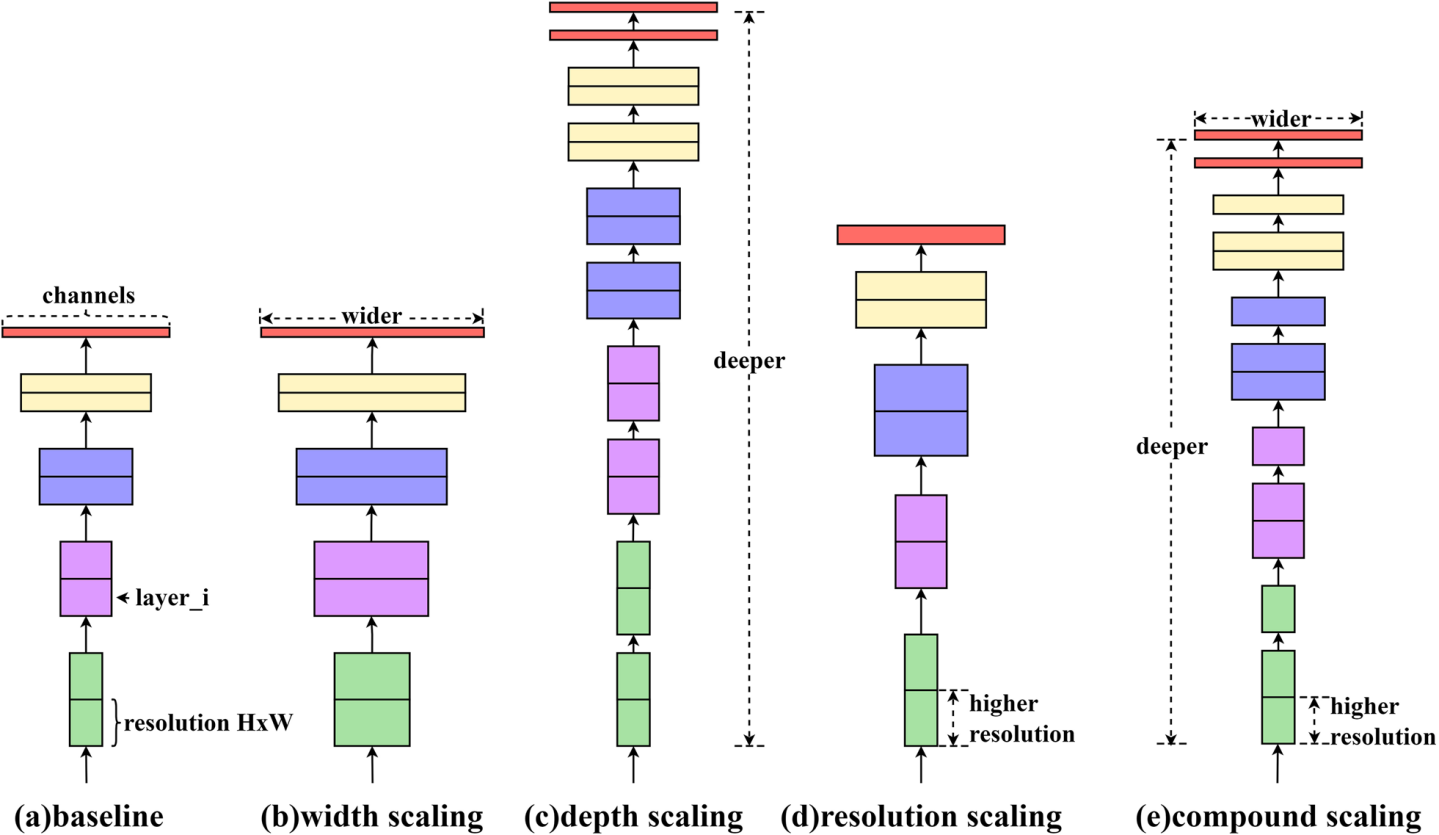
Convolutional neural network model scaling method. **a** Baseline network; **b**–**d** Conventional scaling that only increases one dimension of network width, depth, or resolution; **e** Compound scaling method that uniformly scales all three dimensions with a fixed ratio

When improving the performance of a convolutional neural network, we usually expand the network, generally by adjusting the depth, width and input resolution of the network. For example, ResNet [36] extended ResNet-18 to ResNet-200 by increasing the number of layers. GPipe [37] achieved 84.3% accuracy on ImageNet by extending the CNN baseline 4 times, and the VGG [38] network adopted stacking convolutional blocks to deepen the number of network layers. EfficientNet [39] is a method of mixing model scales including depth, width and resolution of the convolutional network, which can be balanced and adjusted by setting certain parameter values to achieve the best performance. Besides EfficientNet-B7 achieved state-of-the-art 84.4% top-1 accuracy and 97.1% top-5 accuracy on ImageNet, compared with the previous best convolutional network (GPipe, Top-1: 84.3%, Top-5: 97.0%), while the model size is 8.4 times smaller and model speed is 6.1 times faster. The EfficientNet is unlike ResNet and SENet [40], which invented the shortcut or attention mechanism, and the base structure of EfficientNet is established by structure search, and then scaled by compound scaling rules to obtain a series of excellent networks: B0 ~ B7. The CNN model scaling method was shown as Fig. 4, The baseline network (Fig. 4a) was expanded the width, depth, or input resolution as shown in Fig. 4b–d, respectively, and Fig. 4e combined width, depth and input resolution of the network. The EfficientNet has balanced the classification, accuracy and efficiency, so this network was used for image feature extraction.

**Fig. 5:**
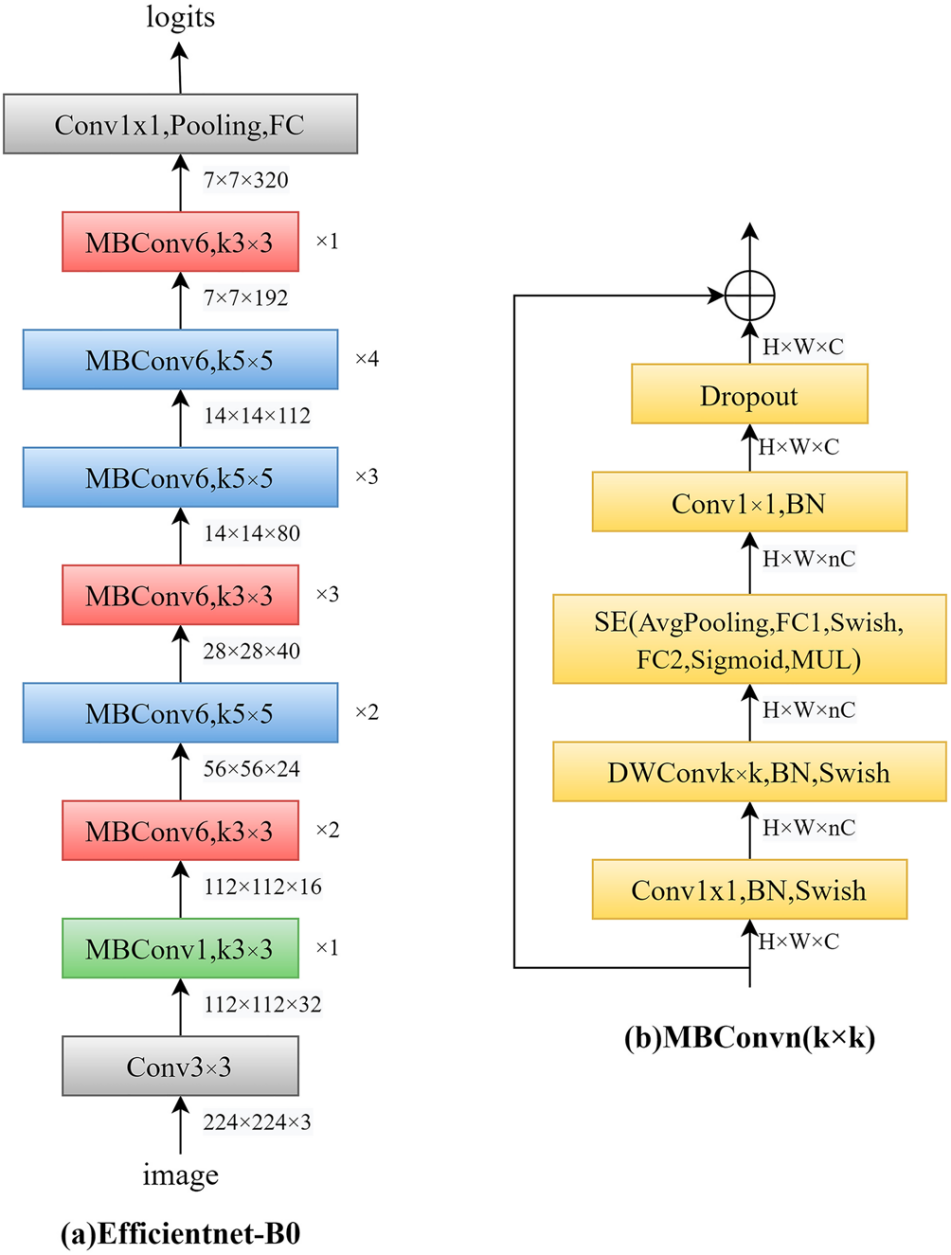
EfficientNet-B0 structure diagram. **a** Overall structure of the network; **b** MBConv structure. MBConv is mobile inverted bottleneck conv, DWConv is depthwise conv, k3 × 3/k5 × 5 is kernel size, BN is batch norm, H × W × C denotes tensor shape (height, width, depth), and × 1/2/3/4 denotes the number of repeated layers within the block

The EfficientNet-B0 structure diagram was shown as Fig. 5, which was divided into 9 stages (Fig. 5a). The first stage is an ordinary convolutional layer (including BN and activation function Swish) with a convolution kernel size of 3 × 3 and a stride of 2. Stage2 ~ Stage8 are repeating the stacking of the MBConv structure by given number. Stage9 consists of an ordinary 1 × 1 convolutional layer (including BN and activation function Swish), an average pooling layer and a fully connected layer. Each MBConv will be followed by the magnification factor n, and the first 1 × 1 convolutional layer in MBConv will expand the channels of the input feature matrix to n times, where k3 × 3 or k5 × 5 represents the size of the convolution kernel used by depthwise (DW) Conv in MBConv. EfficientNet-B1 ~ B7 is to modify the size of the feature matrix (H × W × C) and layers on the basis of B0. Figure 5b showed that the MBConv structure is mainly composed of 1 × 1 ordinary convolution (dimension-raising effect, including BN and Swish), 3 × 3 or 5 × 5 DW Conv (including BN and Swish), SE module, 1 × 1 ordinary convolution (dimension reduction, including BN), and Dropout layer. In the study, we adopted the EfficientNet-B3 for image classification and image feature extraction.

The EfficientNet-B3 was built based on Pytorch1.7.1 and cuda11.0. Based on the previous divided dataset with 499 training sets and 498 test sets, the learning rate was set to 0.01, and the training batch size was set to 32, while the number of training rounds was set to 30. Finally, the classification accuracy of the test set was used as the evaluation index, and the model file was saved. With the trained model, the forward function in the EfficientNet was used to extract the output of the pooling layer as image feature, which was a set of one-dimensional vectors. In order to balance the ratio of spectral features and image features, the output channel number of EfficientNet-B3 was modified according to the number of feature bands. Finally, the image features of cotton leaves were extracted based on the modified EfficientNet.

### Machine learning classification based on spectral and image feature fusion

After the spectral feature bands and image features were extracted individually, they were fused into a set of one-dimensional feature vectors, and normalization was conducted to achieve feature fusion (FF). In this study, the machine learning algorithms including support vector machines (SVM) [41] and back propagation neural networks (BPNN) [42] were adopted to establish classification model of cotton verticillium wilt based on the spectral full band, spectral feature bands and fused features respectively, and the classification performance of the machine learning method was evaluated. The training set and the test set were divided by random method according to the ratio of 5:5, and the 997 total samples were divided into 499 training sets and 498 test sets, while the classification accuracy of the test set was used as the model evaluation index.

SVM is a nonlinear model, which can effectively avoid the dimensional disaster of the sample space, and has the advantages of high precision, fast operation speed, and strong generalization ability [43]. This method has strong advantages in qualitative analysis of a large number of high-dimensional nonlinear hyperspectral data. The purpose of the SVM algorithm is to find the optimal classification hyperplane that maximizes the separation of positive and negative samples on the feature space [44]. When using SVM to establish a classification model, it is necessary to select an appropriate kernel function. There is a lot of application experience that the radial basis function RBF has a good learning ability, and the RBF kernel function as a nonlinear function can reduce the computational complexity in the training process. BPNN is an excellent artificial neural network algorithm, widely used in agriculture. The number of neurons in the input layer is usually related to the number of features, while the number of output layers is defined by the number of categories, and the number of layers and neurons in the hidden layer can be customized [45]. Each neuron represents one processing of the data, the functional relationship between the output and input of each hidden layer and output layer neuron is as Eq. (3–4).

3 $${I}_{j}=\sum_{i}{W}_{ij}{O}_{i}$$

4 $${O}_{j}=sigmod\left({I}_{l}\right)=\frac{1}{1+{e}^{-{I}_{l}}}$$

where ${W}_{ij}$ is the weight of the connection between neuron *i* and neuron *j*; ${O}_{j}$ is the output of neuron *j*; *sigmod* is an activation function of the neuron used to realize nonlinear transformation.

## Result and discussion

### Spectral analysis results

**Fig. 6:**
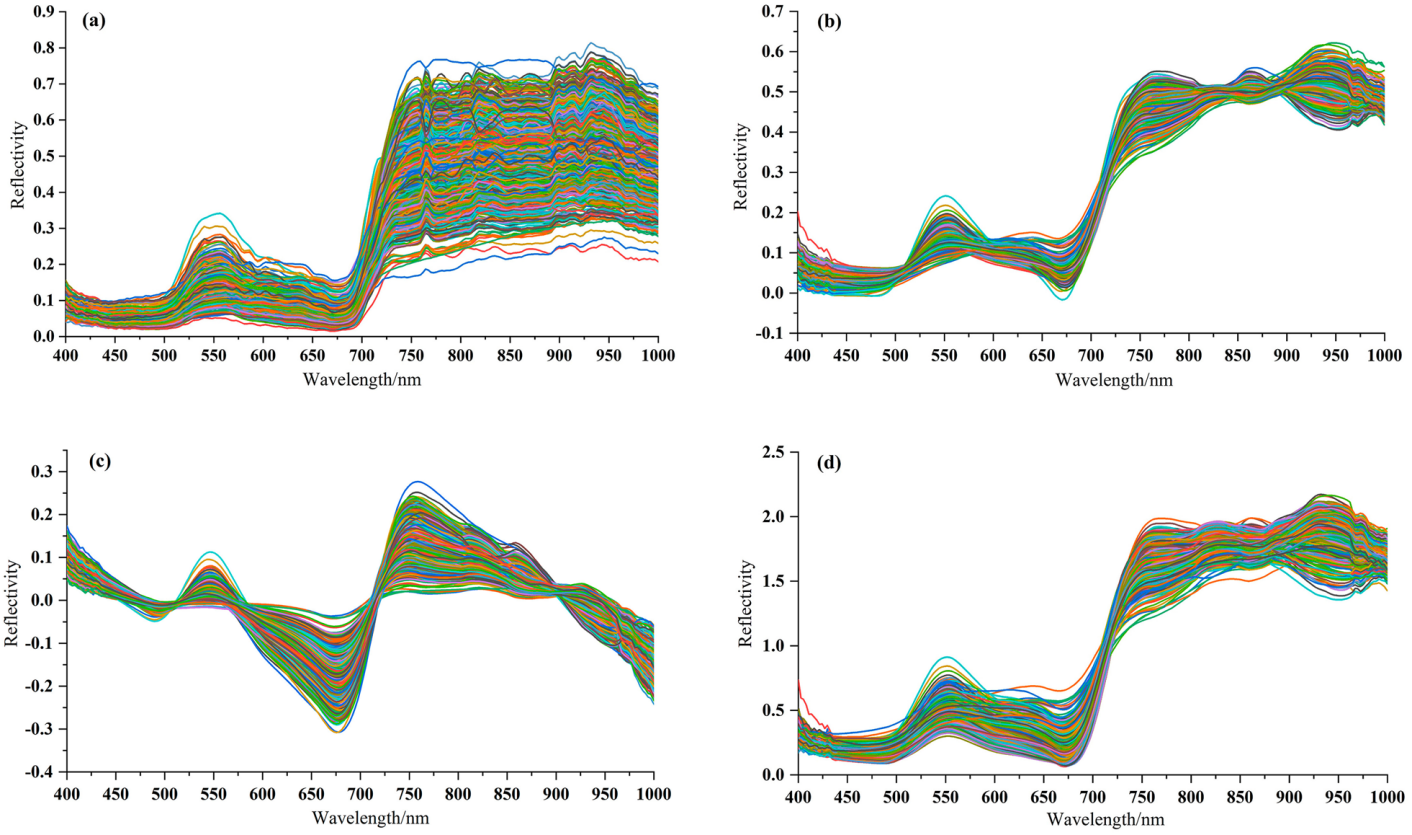
Curve plots of four spectra of 997 cotton leaf samples: **a** original spectra, **b** SG-MSC preprocessed spectra, **c** SG-DT preprocessed spectra, **d** SG-MN preprocessed spectra

**Fig. 7:**
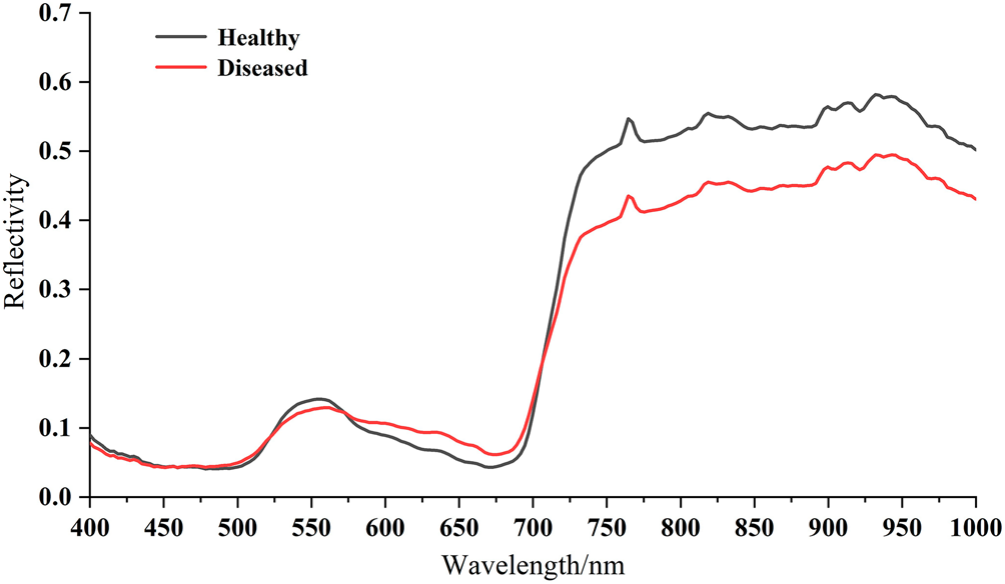
Average spectral curves of healthy leaves and diseased leaves

The spectral curve of 997 cotton leaf samples were shown as Fig. 6, in which the original spectral curve, SG-MSC spectra, SG-DT spectra, and SG-MN spectra were shown as Fig. 6a–d respectively. After preprocessing, the problems including white noise, and baseline drift were significantly reduced. The average spectral curve of healthy leaves and diseased leaves were shown as Fig. 7, and the result proved that the spectral reflectance was low in the blue light band of 400 ~ 500 nm. A small reflectance peak appeared in the band around 550 nm, which was proved to be nitrogen absorption band [46]; a trough appeared in the red band around 680 nm, which was caused by the strong absorption of chlorophyll, and rise significantly from about 700–750 nm, which was caused by the internal light scattering by leaf cells [47]; the spectral band from 730 to 1000 nm was the region of high reflectivity. The spectral curves of healthy leaves and diseased leaves showed typical green leaf characteristics [48], which had the same change trend and high similarity, but there were also significant differences in reflectivity values, which provided data support for subsequent feature extraction and modelling.

### Feature band extraction results

**Fig. 8:**
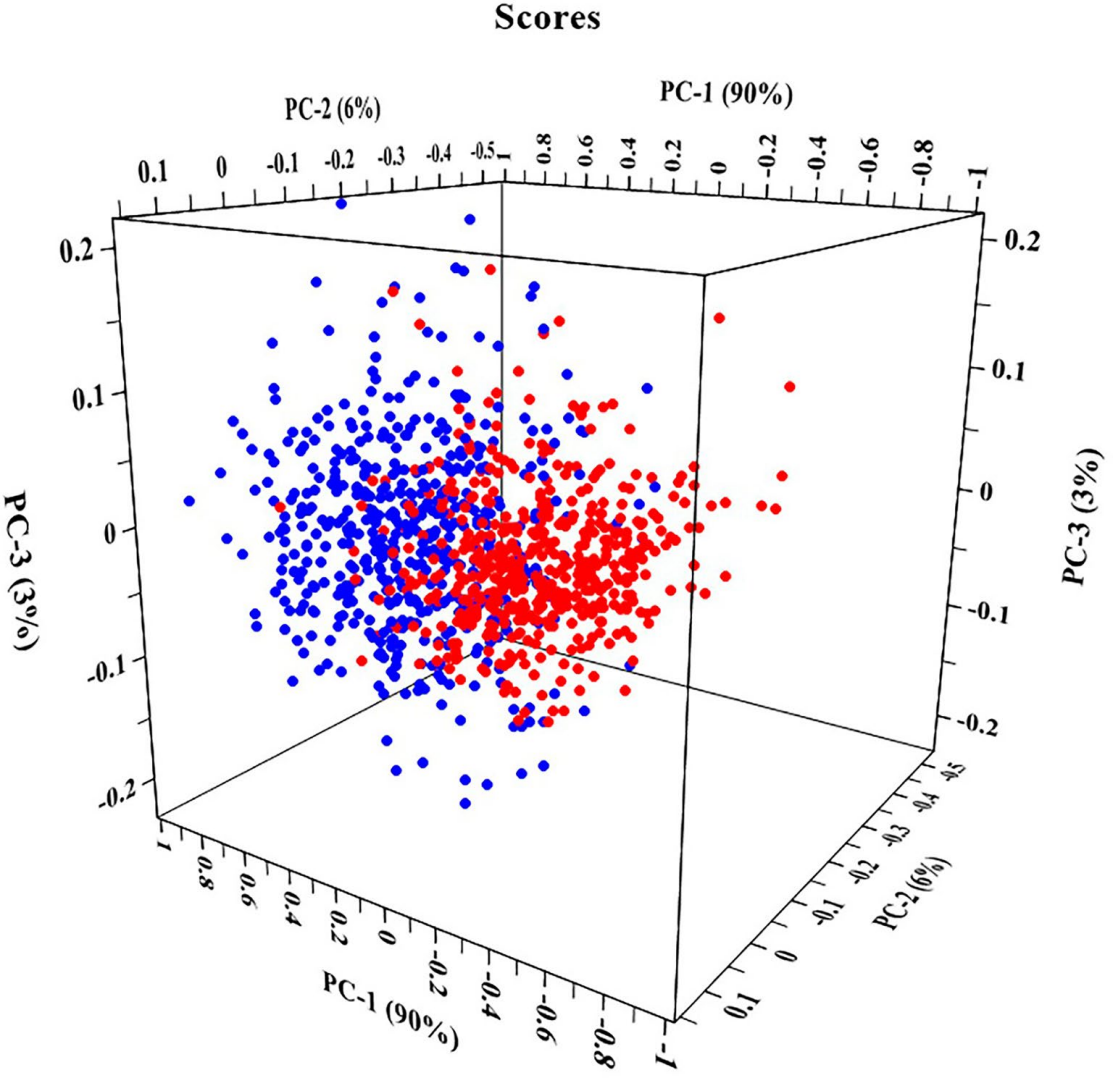
Scores scatter plots of PCA of SG-DT preprocessed spectra. Blue points: scatter points of healthy leaves; Red points: scatter points of diseased leaves

In the study, PCA and SPA algorithm were used to extract feature bands of spectral data, in which 400 ~ 1000 nm were divided into 224 bands. After the PCA was conducted, the principal component loadings coefficient method was applied to select the feature bands, which was computed by the correlation coefficient between the principal component (PC) and the original band variable. Since each PC score was a linear combination by each spectral point multiplied by the corresponding loading, the bands located at local maximal values or minimal values of the loadings curve had a more important contribution to the PC [49]. When the load value was at the peak or trough position of PC load curve, the corresponding band was the feature band [50]. The PCA results of the original spectrum showed that the explained variance of the first three PCs reached 98.97%, which could well express the original variable information. Meanwhile, the explained variance of the first three PCs of the SG-MSC spectra, SG-DT spectra, SG-MN spectra was 93.33%, 98.26%, and 95.48% respectively. The scatter plot of the first three PCs of the SG-DT spectra was showed as Fig. 8, while the blue points were the scatter points of healthy leaves, and the red points are the scatter points of diseased leaves, which proved that there was an obvious contrast between healthy leaves and diseased leaves, so it was feasible to use the spectral data after PCA for modelling and classification. The above method was also applied to process the original spectral data and the preprocessed spectral data by SG-MSC and SG-MN, while the similar results were obtained as Additional file 1: Fig S1.

**Fig. 9:**
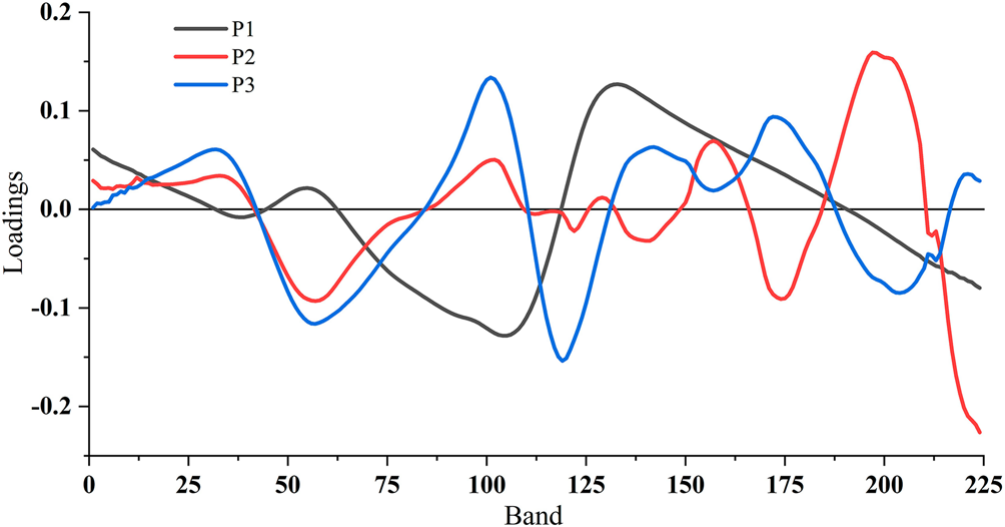
The first three PC load curves of SG-DT preprocessed spectra

The loading curves of the first three PCs of the SG-DT preprocessed spectra were shown as Fig. 9, in which the 15 feature bands were selected from total 224 bands by the principal component loading coefficient method. The loading curves of the first three PCs of the other three preprocessed spectra were shown in Additional file 1: Fig S2. In the same way, the 12 feature bands of the original spectra were obtained, and the 16 feature bands of the SG-MSC spectra and SG-MN spectra were obtained. The feature bands selected by the PCA algorithm were shown in Table 1, which showed that the extracted feature bands were relatively evenly distributed in each interval of the whole band. The results showed that the feature bands could characterize the whole band spectral data.

**Table 1:** Feature bands obtained by different preprocessing methods and different feature extraction algorithms

FBEA	PM	SFB	Selected feature bands
PCA	Original data	12	Band53, Band105, Band111, Band114, Band124, Band132, Band136, Band141, Band156, Band166, Band199, Band221
	SG-MSC	16	Band34, Band 56, Band101, Band104, Band116, Band120, Band132, Band135, Band142, Band156, Band171, Band173, Band203, Band211, Band213, Band220
	SG-DT	15	Band33, Band38, Band56, Band101, Band105, Band119, Band132, Band141, Band157, Band172, Band174, Band197, Band204, Band213, Band221
	SG-MN	16	Band32, Band 36, Band57, Band101, Band104, Band109, Band119, Band133, Band135, Band141, Band157, Band161, Band171, Band174, Band203, Band213
SPA	Original data	10	Band18, Band47, Band73, Band92, Band98, Band105, Band109, Band114, Band135, Band176
	SG-MSC	13	Band3, Band4, Band7, Band9, Band12, Band41, Band67, Band89, Band161, Band177, Band185, Band221, Band224
	SG-DT	7	Band2, Band9, Band12, Band30, Band57, Band102, Band118
	SG-MN	9	Band4, Band6, Band11, Band30, Band56, Band76, Band101, Band120, Band138

*FBEA* feature band extraction algorithm, *PM* preprocessing method, *SFB* number of selected feature bands

**Fig. 10:**
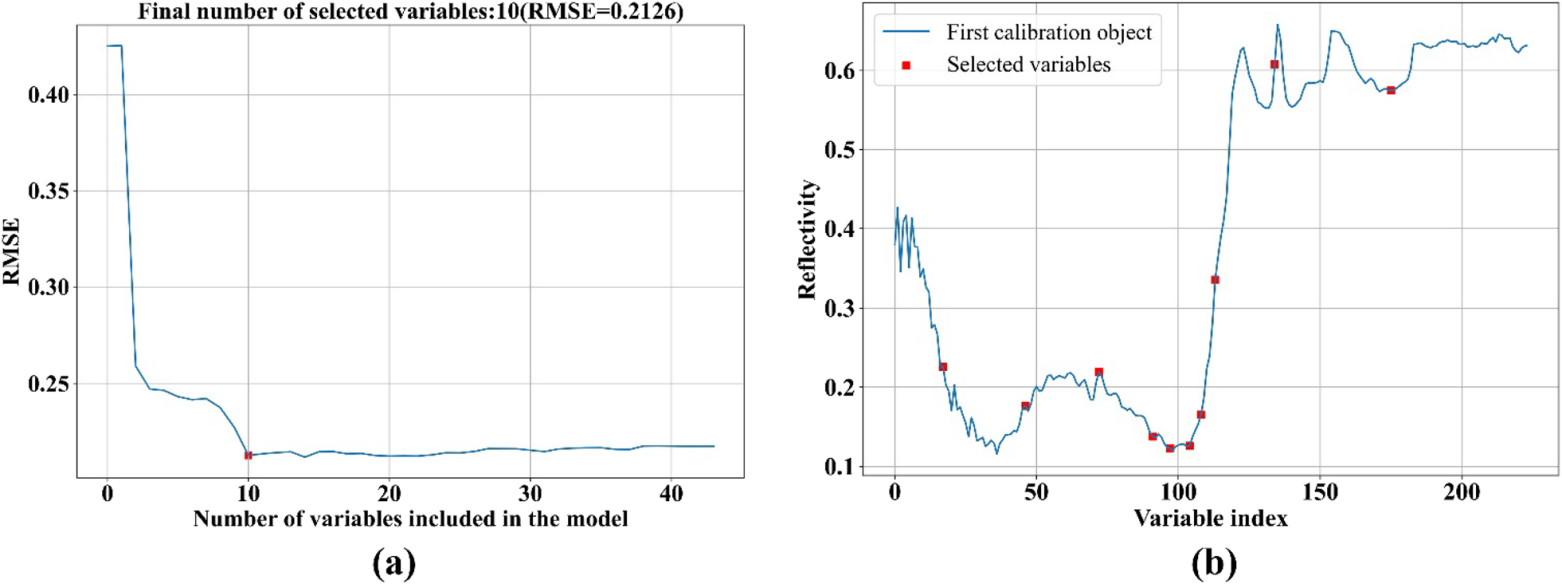
Selection process of feature bands for original spectra using SPA. **a** RMSE screen plot for determining the number of feature bands; **b** Distribution of feature bands marked by each red dot

As the comparison, the successive projections algorithm (SPA) was also adopted to extract the feature bands, and the original spectra results were shown in Fig. 10. The RMSE curve (Fig. 10a) showed that the RMSE value was in a state of decline when the number of bands was less than 10, and then tended to be stable. When the number of feature bands was 10, the RMSE reached minimum value 0.2126, so 10 feature bands were selected, the distribution of which were shown as Fig. 10b. By using SPA, the feature bands for other three preprocessed spectra were shown in Additional file 1: Fig S3. In the same way, 13 feature bands were obtained from SG-MSC spectra, 7 feature bands from SG-DT spectra, and 9 feature bands from SG-MN spectra. The feature bands selected by SPA were shown in Table 1, from the results, the feature bands extracted by the SPA algorithm on the SG-DT spectra were in the forward section, and the number of bands was small, which would lead to low accuracy, and comprehensive features should be further extracted.

In this study, the distance between adjacent bands was found to be 2.7 nm. Using the PCA method, it was discovered that the sensitive bands could be easily identified from the selected feature bands [51]. Each PC load curve differed, with two adjacent peaks and valleys, resulting in a similar phenomenon in the selected feature bands. Additionally, the spectral curve of this study showed that the full band spectra and corresponding feature band curve were similar and displayed the same trend, indicating that the feature band could replace the entire spectrum. Removing some closely-spaced bands would negatively impact feature expression and accuracy, as observed in many studies [52, 53]. The nearest wavelength bands selected by the PCA method in this study differed by 5.4 nm. SPA was a forward selection method, which would add a new band at each iteration until the optimal number N of wavelengths was reached. The N value was determined based on the internal root mean square error (RMSE) value of the calibration set. The set of least-correlated variables might not always yield the best models, and collinearity issues were only indirectly considered in the selection process. SPA would conduct a compromise between these two factors, which aimed to minimize collinearity while utilizing the RMSE criterion to determine the starting point and the optimal number of wavelengths [34]. The results revealed that the feature bands selected by SPA were very close, reflecting a trade-off to achieve the minimum RMSE.

### Image feature extraction based on EfficientNet

**Fig. 11:**
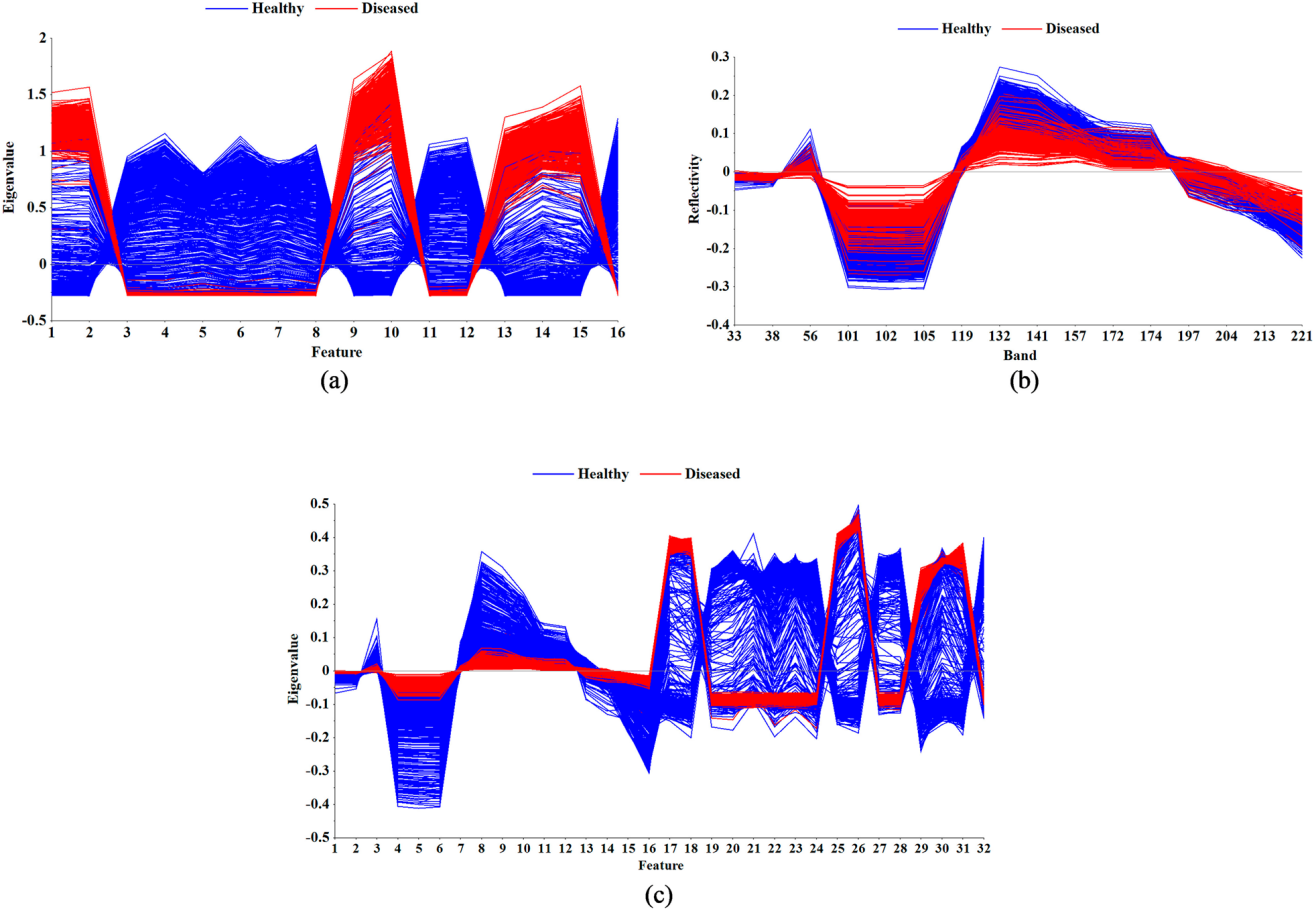
Three kinds of feature curves of SG-DT-PCA method. **a** Graph of image features; **b** Graph of feature bands; **c** Graph of fusion features

The EfficientNet-B3 was adopted for healthy and diseased leaves RGB image classification, and the output channels of the last convolutional layer of the network was adjusted to 16 corresponding with the spectral feature bands number. Then the modified EfficientNet-B3 was trained based on the training set, which would extract the colour and texture features of the RGB image effectively, and the classification accuracy of the test set was 93.00%. Compared with the classification based on spectral features, the classification based on the RGB image features could achieve approximate accuracy. However, there were significant differences between spectral features and image features, so it was possible to improve the classification accuracy by fusing both features. Therefore, the forward function in the EfficientNet was used to extract the output of the pooling layer as the image feature of cotton leaf, which were 997 one-dimensional vectors with 16 elements, and the feature vector curve was shown in Fig. 11a.

### Classification results based on spectral full band

The original spectra were processed using SG-MSC, SG-DT and SG-MN algorithms, and then SVM classification model and BPNN classification model were established. The results were shown in Table 2, in which all models were above 90.00%. By original spectral data, the classification accuracy of the SVM and BPNN model was 94.00% and 94.40% for training set, 91.17% and 92.57% for test set, respectively. By preprocessed spectral data, the optimal classification model in the SVM model was SG-MN-SVM, and the classification accuracy was 95.99% and 92.37% for training set and test set, respectively; the optimal classification model in the BPNN model was SG-MSC-BPNN, and the classification accuracy was 94.19% and 93.78% for training set and test set, respectively. In summary, SG-MSC-BPNN was the optimal classification model among all models. Besides, the results also proved that the BPNN classification model had a better performance than the SVM. But the spectral full band was too large, so it was necessary to refine the spectral bands for classification.

**Table 2:** Results of classification models for healthy and diseased leaves using full band spectral data

Model	Preprocessing method	Classification accuracy (%)	
		Training set	Test set
SVM	Original data	94.00	91.17
	SG-MSC	94.99	92.17
	SG-DT	91.38	91.17
	SG-MN	95.99	92.37
BPNN	Original data	94.40	92.57
	SG-MSC	94.19	93.78
	SG-DT	94.00	93.17
	SG-MN	93.59	93.57

### Classification results based on spectral feature bands

The feature band data selected by PCA and SPA were input into the SVM and BPNN classification models, and the classification results were shown in Table 3. The classification results of the SVM model showed that the accuracy of the training set and test set of the model was 87.58–95.79% and 85.74–93.37% respectively for different feature bands. And the classification results of the BPNN model showed that the accuracy of the training set and test set of the model was 91.18–95.60% and 89.96–93.78% for different feature bands respectively. Among the established SVM models, the SG-MN-SPA feature bands had the best classification performance, while the accuracy of the training set and test set was 95.79% and 93.37%, respectively. Among the established BPNN models, the SG-MN-SPA feature bands had the best results, while the accuracy of the training set and test set was 95.60% and 93.78% respectively. In conclusion, the SG-MN-SPA-BPNN model had the best classification performance in this study, which outperformed the SG-MN spectral full band model with an increase of 0.21%. However, the model was still needed to be improved, before the practical application of cotton verticillium wilt identification, so the morphological and color features based on RGB image were extracted to achieve better classification results.

**Table 3:** Results of classification models for healthy and diseased leaves using feature band data

Feature band extraction algorithm	Preprocessing method	SVM classification accuracy (%)		BPNN classification accuracy (%)	
		Training set	Test set	Training set	Test set
PCA	Original data	91.78	89.16	94.19	91.17
	SG-MSC	90.58	89.36	94.19	92.37
	SG-DT	87.58	86.15	92.39	91.57
	SG-MN	93.79	90.96	93.19	92.97
SPA	Original data	94.59	92.77	94.00	91.77
	SG-MSC	92.79	91.57	92.18	90.56
	SG-DT	88.38	85.74	91.18	89.96
	SG-MN	95.79	93.37	95.60	93.78

### Classification results based on spectral and image feature fusion

The feature bands extracted in Table 1 were respectively fused with image features, and the fusion feature curve of SG-DT-PCA-FF was shown in Fig. 11c. Compared with the SG-DT-PCA feature bands (Fig. 11b), it was obvious that the fusion features had greater differentiation, and the fusion features of the other methods had similar results, which were shown in Additional file 1: Fig S4 and Additional file 1: Fig S5, indicating that the fusion features had better characterization ability. Then the fusion features were input into the SVM and BPNN classification models, and the classification results were shown in Table 4. The results showed that the classification accuracy of all models had been significantly improved. As to the SVM model, the accuracy of the training set and test set of the model was 96.59–98.39% and 96.19–97.99%, respectively; As to the BPNN model, the accuracy of the training set and test set of the model was 97.40–99.20% and 96.59–98.99%, respectively. Especially in the SG-DT-PCA-FF-SVM and SG-DT-SPA-FF-SVM models, the fusion feature significantly improved the accuracy by 10.65% and 10.45% respectively. Among the established SVM models, the SG-MN-PCA-FF-SVM model had the best classification effect, and the classification accuracy of its training set and test set was 98.20% and 97.99%, respectively. Among the established BPNN models, the SG-MN-SPA-FF-BPNN model had the best classification effect, and the classification accuracy of its training set and test set was 99.00% and 98.99%, respectively, which outperformed all the SVM models. The results demonstrated that the fusion of spectral and image features could significantly improve the classification performance, and the classification accuracy of the optimal model had reached 98.99%, which had a very considerable classification effect.

**Table 4:** Results of classification models for healthy and diseased leaves using fused feature data

Feature band extraction algorithm	Fusion method	SVM classification accuracy (%)		BPNN classification accuracy (%)	
		Training set	Test set	Training set	Test set
PCA	Original data-FF	97.80	97.00	97.60	97.19
	SG-MSC-FF	98.00	97.59	99.20	98.59
	SG-DT-FF	97.19	96.79	98.60	97.39
	SG-MN-FF	98.20	97.99	98.59	98.39
SPA	Original data-FF	96.99	96.59	98.60	97.79
	SG-MSC-FF	98.20	97.60	98.80	98.19
	SG-DT-FF	96.59	96.19	97.40	96.59
	SG-MN-FF	98.39	97.79	99.00	98.99

## Conclusion

This study has demonstrated a novel method for cotton verticillium wilt identification based on spectral and image feature fusion, which obviously outperformed than the classification method solely based on spectral features or image features. In the research, the preprocessing methods including SG, MSC, DT and MN, and the feature bands extraction methods including PCA and SPA, were studied to obtain the optimal technical route of hyperspectral data analysis for cotton verticillium wilt identification. Meanwhile, the modified EfficientNet was adopted to identify the healthy and diseased leaves, while the image features were extracted. Then, SVM and BPNN models were established based on the spectral full band, spectral feature bands and fused features, respectively. Finally, the following conclusions and prospects were drawn.

A) As to the spectral full bands, SG-MSC-BPNN model had the better performance with the test accuracy of 93.78%, which proved that the preprocessing method of SG and MSC could improve the model accuracy, and the BPNN model was better than SVM. As to the spectral feature bands, SG-MN-SPA-BPNN model obtained the best classification accuracy of 93.78% in test set. The feature band extraction algorithms could effectively reduce the data dimension, which would promote the model generalization, but decrease the test accuracy.
B) As to the EfficientNet, the classification accuracy of healthy and diseased leaves was 93.00%, which could extract the leaf shape and color features effectively. As to the fused features, SG-MN-SPA-FF-BPNN model obtained the best performance with the test accuracy of 98.99%. Compared with spectral or image features, the fused feature could significantly improve the model accuracy.
C) This study proved an objective and efficient method for cotton verticillium wilt identification, and even achieved higher accuracy, which would promote the researchers to explore the relationship between the spectral and image features with the cotton verticillium wilt. And it would provided a novel method for cotton breeding and disease resistance research. Moreover, the early identification and dynamic detection of cotton verticillium wilt would be feasible based on spectral and image feature fusion, in the future research.

## Supplementary Information

**Additional file 1: Figure S1.** Scores scatter plots of PCA of other three different preprocessing spectra: (a) original data, (b) SG-MSC, (c) SG-MN. Blue points: scatter points of healthy leaves; Red points: scatter points of diseased leaves. **Figure S2.** The first three PC load curves of other three different preprocessing spectra: (a) original data, (b) SG-MSC, (c) SG-MN. **Figure S3.** Selection process of characteristic bands using SPA. RMSE screen plot for determining the number of characteristic bands; SPA screen plot shows distribution of characteristic bands marked by each red dot. **Figure S4**. Characteristic band curves using the other seven methods. **Figure S5.** Fusion feature curves using the other seven methods.
