# Supplementary material for: Intelligent identification on cotton verticillium wilt based on spectral and image feature fusion
Source: Plant Methods. 2023 Jul 29;19:75. doi: 10.1186/s13007-023-01056-4 (PMC10385904; doi:10.1186/s13007-023-01056-4)
Supplement: Supplementary file 1 — Additional file 1: Figure S1. Scores scatter plots of PCA of other three different preprocessing spectra: (a) original data, (b) SG-MSC, (c) SG-MN. Blue points: scatter points of healthy leaves; Red points: scatter points of diseased leaves. Figure S2. The first three PC load curves of other three different preprocessing spectra: (a) original data, (b) SG-MSC, (c) SG-MN. Figure S3. Selection process of characteristic bands using SPA. RMSE screen plot for determining the number of characteristic bands; SPA screen plot shows distribution of characteristic bands marked by each red dot. Figure S4. Characteristic band curves using the other seven methods. Figure S5. Fusion feature curves using the other seven methods. [file 13007_2023_1056_MOESM1_ESM.docx]

**Figure. S1.** Scores scatter plots of PCA of other three different preprocessing spectra: (a) original data, (b) SG-MSC, (c) SG-MN. Blue points: scatter points of healthy leaves; Red points: scatter points of diseased leaves.

**Figure. S2.** The first three PC load curves of other three different preprocessing spectra: (a) original data, (b) SG-MSC, (c) SG-MN.


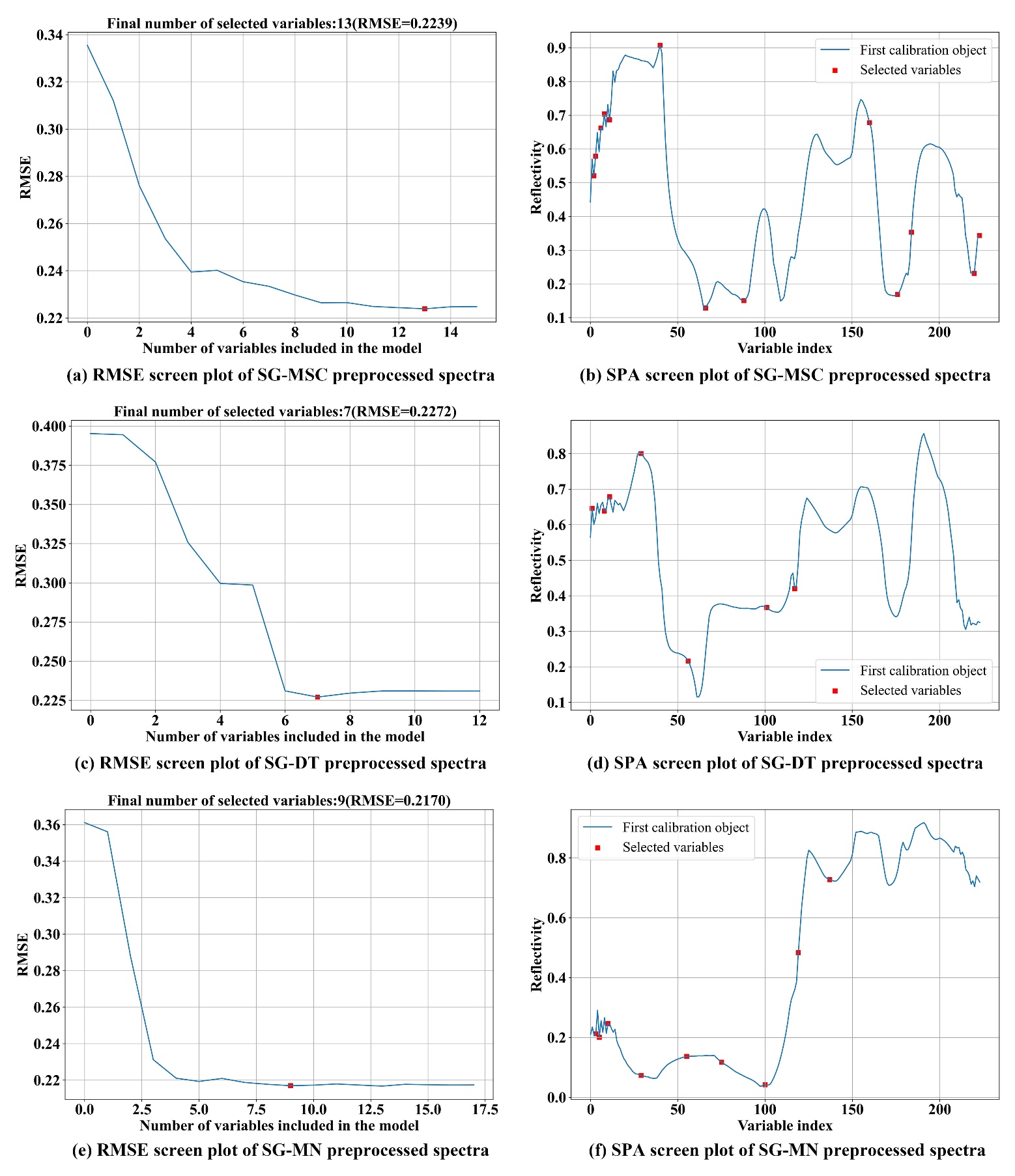


**Figure. S3.** Selection process of characteristic bands using SPA. RMSE screen plot for determining the number of characteristic bands; SPA screen plot shows distribution of characteristic bands marked by each red dot.


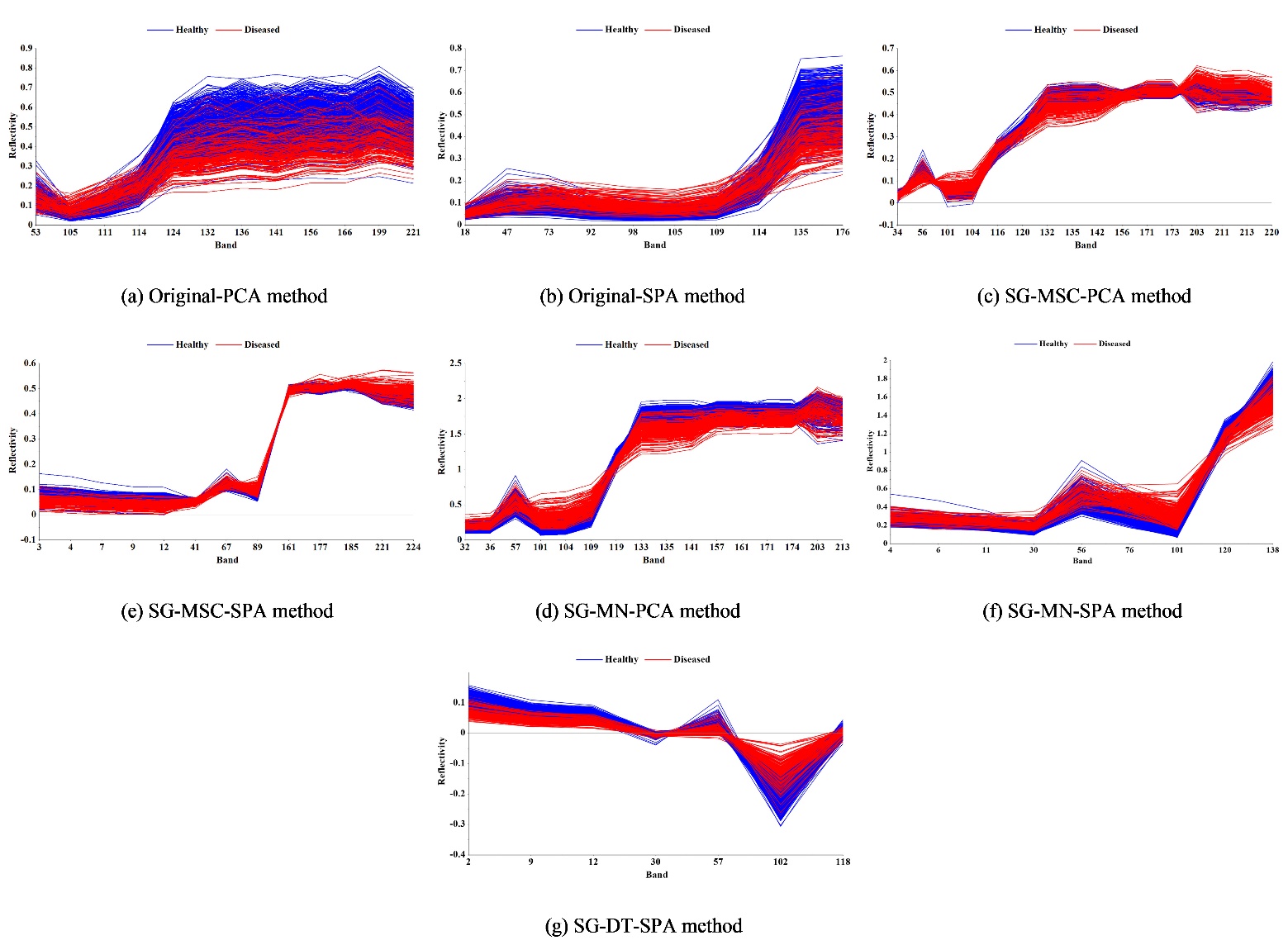


**Figure. S4.** Characteristic band curves using the other seven methods


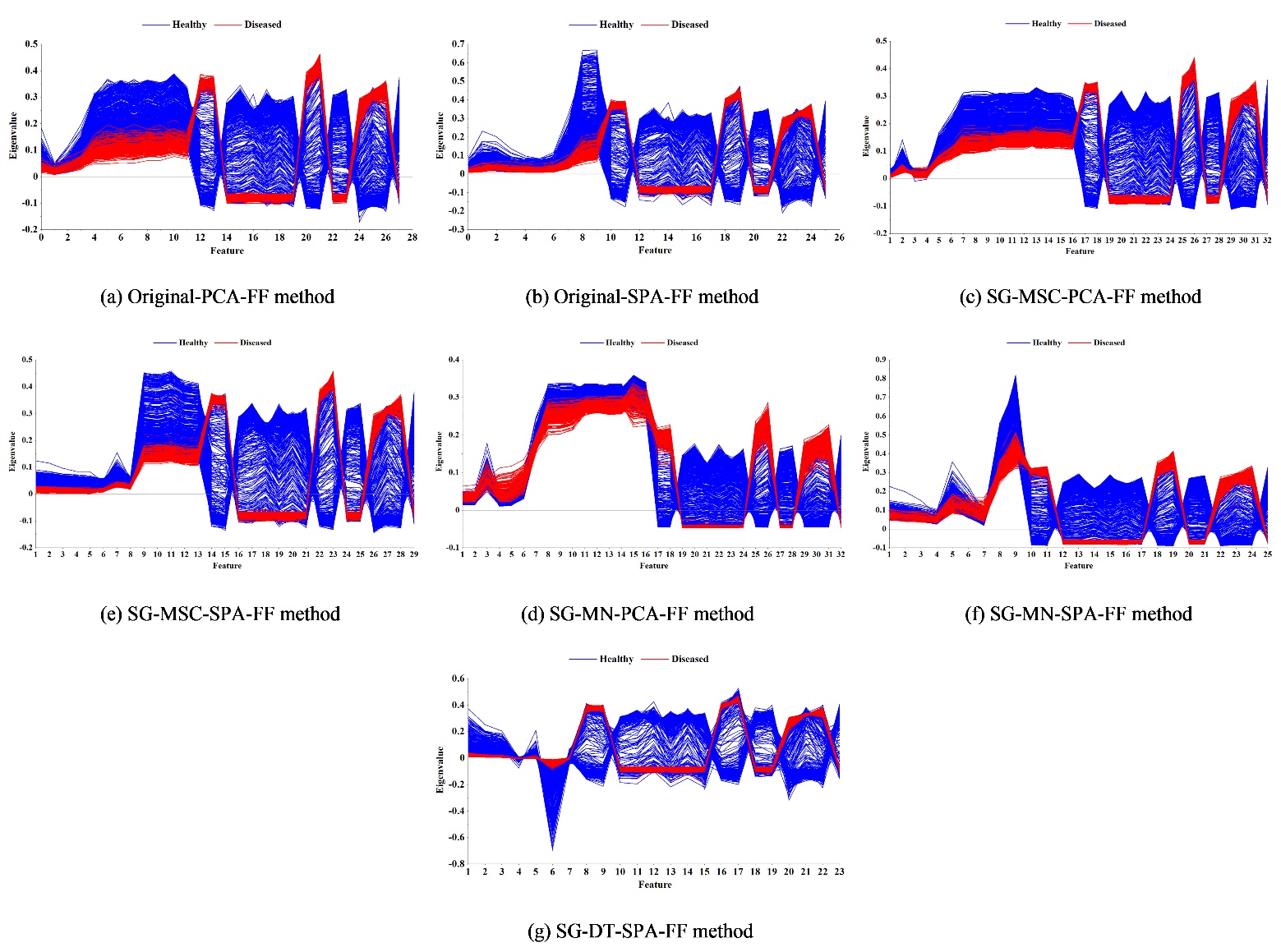


**Figure. S5.** Fusion feature curves using the other seven methods
